# Tau propagation is dependent on the genetic background of mouse strains

**DOI:** 10.1093/braincomms/fcac048

**Published:** 2022-02-23

**Authors:** Simon Dujardin, Analiese Fernandes, Riley Bannon, Caitlin Commins, Mark De Los Santos, Tarun V. Kamath, Mansuo Hayashi, Bradley T. Hyman

**Affiliations:** 1 Department of Neurology, Massachusetts General Hospital, Charlestown, MA, USA; 2 Harvard Medical School, Boston, MA, USA; 3 Eli Lilly and Company, Indianapolis, IN, USA

**Keywords:** tauopathies, Alzheimer’s disease, tau spreading, genetic background, cell-to-cell transfer

## Abstract

Progressive cognitive decline in Alzheimer’s disease correlates closely with the spread of tau protein aggregation across neural networks of the cortical mantle. We tested the hypothesis that heritable factors may influence the rate of propagation of tau pathology across brain regions in a model system, taking advantage of well-defined genetically diverse background strains in mice. We virally expressed human tau locally in the hippocampus and the entorhinal cortex neurons and monitored the cell-to-cell tau protein spread by immunolabelling. Interestingly, some strains showed more tau spreading than others while tau misfolding accumulated at the same rate in all tested mouse strains. Genetic factors may contribute to tau pathology progression across brain networks, which could help refine mechanisms underlying tau cell-to-cell transfer and accumulation, and potentially provide targets for understanding patient-to-patient variability in the rate of disease progression in Alzheimer’s disease.

## Teaser

Dujardin *et al*. report that when human tau is expressed locally in the brain of mice with diverse genetic backgrounds, some backgrounds show more tau cell-to-cell spreading than others while tau misfolding accumulated at the same rate underpinning that genetic factors may contribute to tau pathology progression across brain networks.

## Graphical Abstract

**Graphical Abstract:**
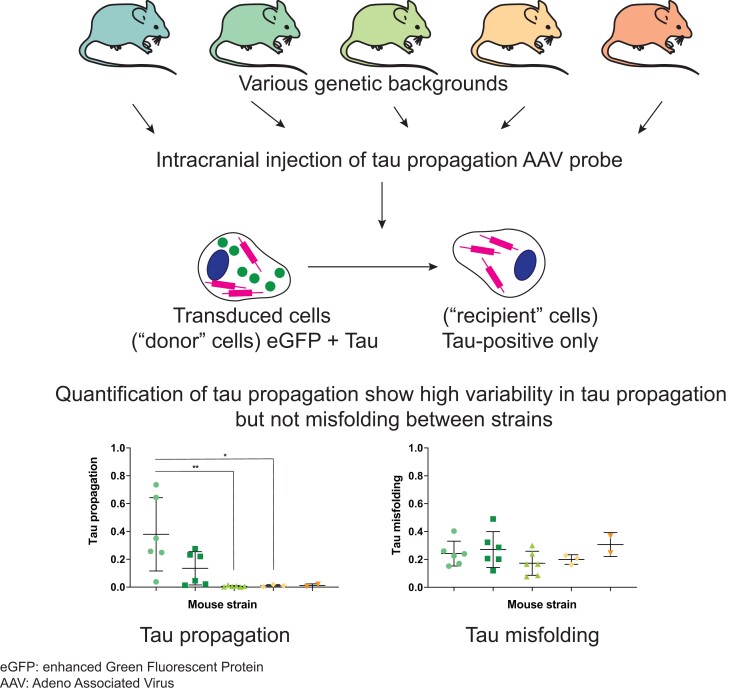

## Introduction

The intraneuronal deposition of hyperphosphorylated tau proteins in insoluble aggregates is a hallmark of around 20 pathologies known as tauopathies including Alzheimer’s disease.^1^ Characteristic populations of neurons in each of these disorders begin to accumulate tau inclusions, which then typically ‘spread’ to anatomically connected sites as the disease progresses, in a process referred to as propagation.^2–4^ Risk factors such as age and genetics clearly contribute to these diseases.^5–8^ We have developed an animal model system to measure the propagation of tau species from one neuron to the next *in vivo*, by transducing neurons with an adeno-associated viral (AAV) vectors construct that encodes both green fluorescent protein (GFP) and human tau. Transduced neurons immunostain for both gene products, whereas a subset of neurons downstream of the injection site contain only tau, and are interpreted to have received human tau protein trans-synaptically.^9,10^ We have previously used this system to demonstrate that age impacts the likelihood of propagation of tau to downstream neurons.^5^ We now have examined a set of genetically diverse mouse strains, to begin to explore the possibility that heritable factors might impact tau propagation.

## Materials and methods

### Animals

All procedures were performed following the guidelines of the Institutional Animal Care and Use Committee and in compliance with the Animal Welfare Act, the Guide for the Care and Use of Laboratory Animals, the Office of Laboratory Animal Welfare and the guidelines of Massachusetts General Hospital. The animals’ living conditions, including housing, feeding and non-medical care, were maintained by the house internal animal facility. Mice of the following strains were purchased from the Jackson Laboratory (Bar Harbor, ME, USA): A/J (Stock Number #000646), C57BL/6J (Stock Number #000664), 129S1/SvlmJ (Stock Number #002448) and CAST/EiJ (Stock Number #000928), CD-1 IGS mice (Strain Code 022) were obtained from Charles River (Wilmington, MA, USA).

### AAV design, cloning and production

The cloning of enhanced GFP (eGFP)-2a-WTtau and eGFP-2a-P301Ltau under the ubiquitous chicken β-actin promoter was performed as described previously.^5^ The plasmid DNA of both eGFP-2a-huTau constructs and GFP were then tested for inverted terminal repeat integrity by digestion with the restriction enzyme Sma I, DNA of sufficient quality was packaged into an AAV2/8 (titres ∼0.6 × 10^13^ virus particles/ml; Massachusetts Eye and Ear Institute vector core), and active AAV stock was aliquoted and stored at −80°C to prevent freeze–thaw cycles.

### Intracranial injections

AAVs encoding eGFP-2a-P301Ltau were injected bilaterally into the entorhinal cortex and in the contralateral hippocampus of 3-month-old mice (per injection site: 2.0 µl of AAV, AAV concentration: 0.6 × 10^13^ virus particles/ml). Six animals per strain were injected, three males and three females. Animals, where the injection sites were missed, were excluded from subsequent analysis resulting in a lower *n* number in some groups. Injections were performed as described previously under standard aseptic surgery conditions: animals were anaesthetized with isoflurane (3% induction and 2% maintenance), a midline incision of the skin was made above the injection sites and burr holes were drilled through the skull at the selected coordinates; coordinates (from bregma) for EC injections: anterior/posterior (A/P): −4.7 mm, medial/lateral (M/L): −4.5 mm, dorsal/ventral (D/V): −2.0 mm from brain surface with an 18° angle; coordinates (from bregma) for the hippocampus injections: A/P: −2.5 mm, M/L: +2 mm, D/V: −2 mm. After lowering the needle into the brain to the injection location, AAV solutions were injected at a flow rate of 0.2 µl/min. A 10 µl Hamilton syringe with a 30 gauge bevelled needle that was coupled to an injector pump was used. The injector was attached to a stereotaxic frame, in which the mice were head fixated. After finishing the injection, the needle was left in place for 2 min to allow the diffusion of the injected AAV solution. Afterwards, the skin over the injection site was sutured, and the animals were allowed to recover from anaesthesia on a 37°C warming pad before returning them into a clean home cage. For analgesia, all mice received a subcutaneous injection of buprenorphine (0.05 mg/kg) immediately after AAV injection and were treated with Tylenol (in drinking water) for 3 days after the surgery.

### Immunofluorescence labelling

For immunofluorescence labelling of brain sections, injected mice were transcardially perfused with cold phosphate-buffered saline (PBS) for 5 min before switching to PBS containing 4% paraformaldehyde. The whole brains were extracted and postfixed in 4% PFA/PBS for 2 days at 4°C and then cryoprotected in 30% (w/v) sucrose in PBS until they sank, cut horizontally into 40 µm-thick brain sections on a freezing microtome and stored in PBS/50% glycerol at −20°C. For immunostaining, the floating brain sections were washed briefly in PBS and then permeabilized with 0.2% Triton X-100/TBS for 20 min at room temperature, blocked in 5% normal goat serum/PBS for 1 h at room temperature and then incubated with primary antibodies diluted in 3% NGS/PBS overnight at 4°C: chicken anti-eGFP (1:1000, Aves Labs), mouse anti-human tau Tau13 (1:1000, BioLegend) and mouse anti misfolded tau Alz50 (1/100, kind gift from Dr Peter Davis). After washing three times with PBS, secondary antibodies were diluted in 3% NGS/PBS and applied for 1.5 h at room temperature: Alexa 488 anti-chicken, Alexa 555 anti-mouse (1:1000, Thermo Fisher Scientific). After three washes in PBS, sections were stained with 4′,6-diamidino-2-phenylindole (DAPI) and then mounted on microscope glass slides with mounting media.

Imaging of immunolabelled sections was done using the 40× objective on an Olympus VS120.

### Stereological cell counts

For the stereological analysis, a series of 40 µm-thick coronal sections separated by 360 µm were analysed. The number of huTau donor cells, huTau recipient cells and DAPI+ nuclei as well as the number of cells positive for misfolded tau, in the EC, subiculum and hippocampal formation of AAV-injected hemispheres were determined by counting all (eGFP+), (huTau+/GFP−), cells in the area of interest using the cellSens software (Olympus). The per cent area of GFP expression was obtained by thresholding using the cellSens software (Olympus).

### Statistical analysis

To compare cell numbers and propagation rates, we determined the average cell number or cell percentage (no. of recipients/no. of donors) per mouse, individual mice being the experimental unit of the analysis in this study. Statistical analysis of differences between groups was performed using GraphPad Prism 6; when normal distribution was applicable, groups were compared using one-way ANOVA Tukey’s test for multiple comparison. Non-parametric distributions were analysed using the Mann–Whitney non-parametric test. All values are given as mean ± standard error of the mean.

### Data availability

All data reported in this manuscript are stored at the Massachusetts General Hospital and are available from the corresponding author upon reasonable request.

## Results

**Figure 1:**
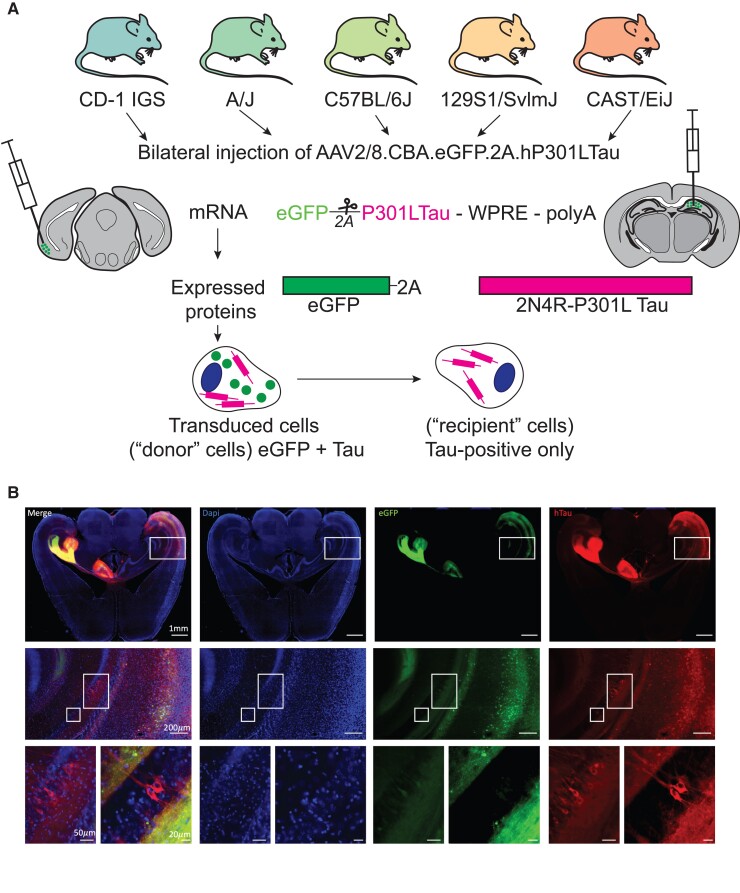
**Model of tau propagation.** (A) Schematic showing the mouse strains used in this study, the AAV sequence, the mRNA and the proteins encoded in AAV CBA.eGFP-2a-P301Ltau, as well as the tau protein propagation principle and detection methodology. Using a self-cleaving 2a peptide, transduced ‘donor’ neurons express both eGFP and human tau as individual proteins. The propagation of tau can be visualized by immunofluorescence labelling of post-mortem brain sections or fixed neurons in culture: human tau detected in ‘recipient’ neurons that do not express the fluorescence transduction marker eGFP indicates the propagation of tau between cells. Thereby, the upstream location of the GFP transduction marker prevents the detection of false positives that could occur due to incomplete translation of the mRNA. Bilateral injection of AAV eGFP-2a-P301LTau into the entorhinal cortex (left side) and in the hippocampus (right side). (**B**) Representative images of immunohistochemistry results and tau propagation recipient neurons at different magnifications showing injection sites (upper pictures), entorhinal cortex to hippocampal formation connection (middle pictures) and two magnifications showing tau propagation recipient neurons in the hippocampus (lower pictures); scale bars are indicated in the figure.

**Figure 2:**
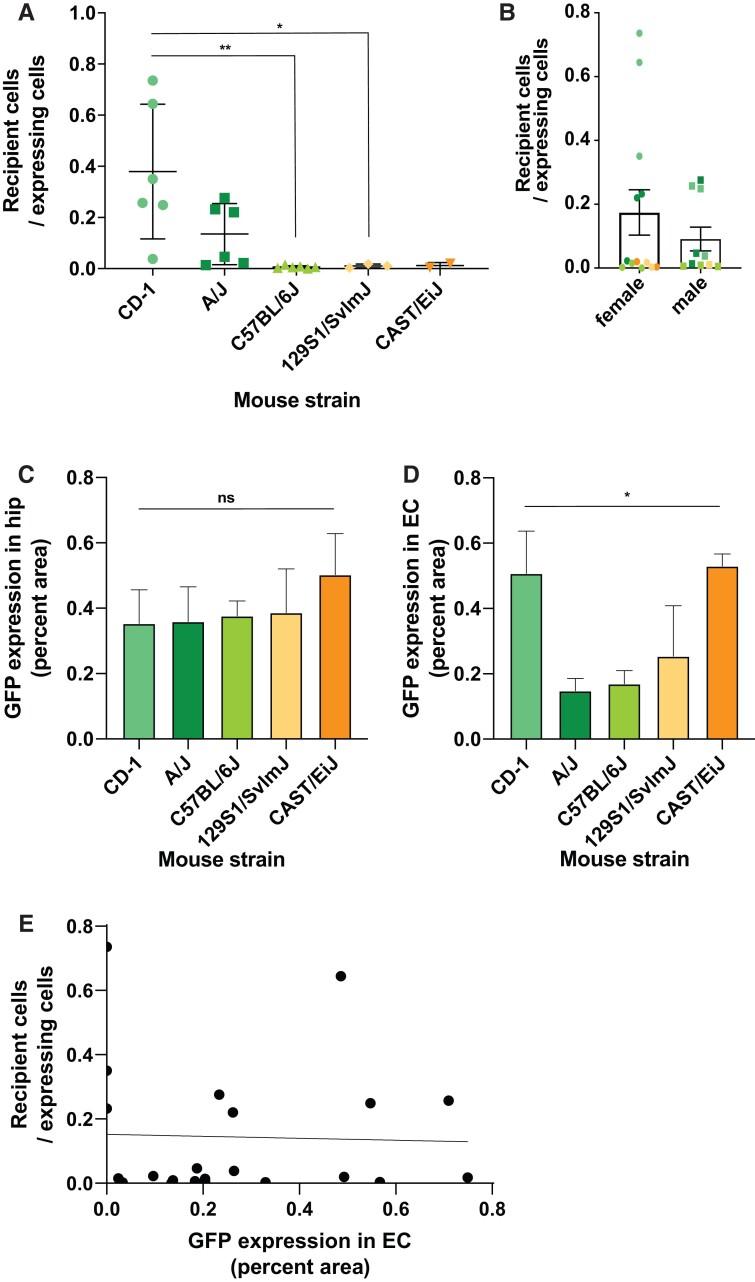
**Tau propagation is dependent on genetic background.** (**A**) Ratio of recipient cells (hTau+, GFP−) to AAVs expressing cells (hTau+, eGFP+) across mouse strains showing significant differences between strains (one-way ANOVA, *P* = 0.0037; with the Tukey multiple comparison test, *P < 0.05, **P < 0.01). (**B**) Ratio of recipient cells (hTau+, eGFP−) to AAVs expressing cells (hTau+, GFP+) in male versus female animals, regardless of the strain. Mann–Whitney non-parametric test, *P* = 0.97. (**C and D**) Burden of eGFP expression at the injection sites in the hippocampus (**C**) and entorhinal cortex (**D**) (one-way ANOVA; *P < 0.05. The Tukey multiple comparison test did not show significant differences between specific groups). (**E**) Tau propagation ratio in the hippocampus versus eGFP expression in the entorhinal cortex shows no association. A non-parametric Pearson test was used for this correlation (*r* = −0.033; *P*-value = 0.88).

We used a model of tau propagation that we previously described in which AAVs encoding the eGFP-2A-Tau construct were injected into the entorhinal cortex or the hippocampus of mice.^5,9–11^ The 2A peptide is a self-cleaving peptide resulting in the equimolar independent expression of the eGFP and human tau (Fig. 1A). Using this model, we can, therefore, use immunostaining to easily discriminate the donor neurons transduced with the AAV (eGFP and tau positive) from the tau propagation recipient neurons (tau positive only) (Fig. 1A and B). In order to understand if genetic background had an effect on tau propagation, we selected five mouse strains genetically distant from each other (129S1/SvlmJ, A/J, C57BL/6J, CAST/EiJ and CD-1) and injected them with the eGFP-2A-Tau AAVs in the CA1 region of the hippocampus on one side and in the entorhinal cortex on the contralateral side (Fig. 1A and B). Twelve weeks post-injection, we collected and immunostained the brains (Fig. 1B). We quantified the number of (i) tau human positive, eGFP-positive neurons (neurons expressing the AAVs, herein referred as ‘expressing neurons’) or (ii) human tau positive, eGFP-negative neurons (recipients of tau propagation, herein referred as ‘recipient neurons’) (Fig. 1B). For each animal, we calculated the ratio of recipient neurons to expressing neurons and observed significant differences among strains, with some strains, such as 129S1/SvlmJ or C57BL/6J, being ‘resilient’ to tau propagation and some strains, A/J and CD-1 in particular, exhibiting consistently increased tau propagation (Figs 1B and 2A).

In a set of secondary analyses, we compared the effect of sex among the whole cohort. There is a slight tendency towards female animals showing more tau propagation, but the difference was not significant (Fig. 2B) and mostly pooled by CD-1 females (SupplementaryFig. 1). We then compared within strains and found that there appeared to be a difference in tau propagation between CD-1 females and CD-1 males. Although this reached statistical significance (*P* = 0.032), we interpret this with caution as the effect was seen in only one strain out of five, and the relatively small group sizes may confound secondary analyses.

We wondered if the difference in propagation between strains could reflect differential transduction or expression of the AAVs in the different strains. We, therefore, quantified the number of AAV-expressing neurons in the two injection sites. In the hippocampus, no apparent difference was seen in the expression of the AAVs (Fig. 2C). More variability is observed in the entorhinal cortex, perhaps due to technical variability in the exact site of injection (Fig. 2D). However, these differences do not seem to account for differences in the amount of tau propagation neurons across species (Fig. 2E), reinforcing the idea that the differences in tau propagation between strains are not due to differential expression of the AAVs.

**Figure 3:**
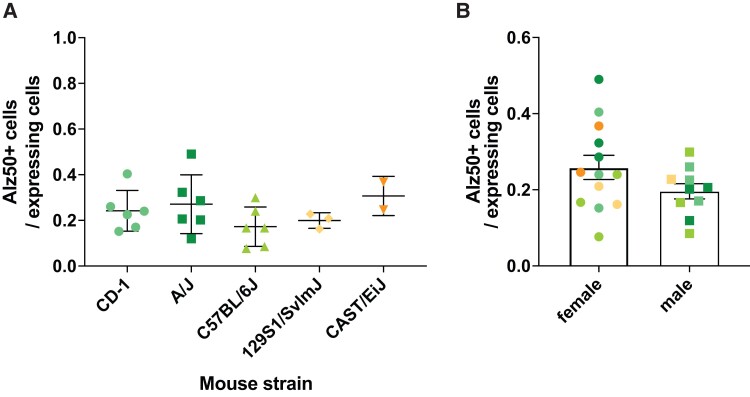
**Misfolded tau is not dependent on genetic background.** (**A**) Ratio of misfolded tau-positive cells (Alz50+) to expressing cells (eGFP+) across mouse strains (one-way ANOVA, *P* = 0.35). (**B**) Ratio of misfolded tau-positive cells (Alz50+) to expressing cells (eGFP+) in male and female animals, regardless of the strain Mann–Whitney non-parametric test, *P* = 0.21.

Viral-mediated overexpression of tau can drive the formation of pathological tau phenotypes such as the appearance of epitopes of tau hyperphosphorylation or tau misfolding.^9,12,13^ We postulated that genetic background in these mice would influence the development of such pathological phenotypes on tau. Therefore, we immunostained the brain sections with an antibody recognizing a misfolded form of tau characteristic of pathological tau (Alz50) and quantified the number of positive neurons. The first observation is that most tau propagation recipient neurons are mostly negative for Alz50 staining and that most of the Alz50+ neurons are eGFP positive. Interestingly, neither genetic background (Fig. 3A) nor sex within strains or across the cohort (Fig. 3B and Supplementary Fig. 1B) seemed to affect misfolding.

## Discussion

Recent data have shown the importance of genetic background in the design of mouse models of Alzheimer’s disease.^14^ Several studies show differences in tau pathology accumulation in the rTg4510 mouse model of tauopathy with different backgrounds.^15,16^ In a tau seeding paradigm in rat transgenic models, Smolek *et al*.^17^ also find genetic background heterogeneity. Interestingly, both amyloid β-deposition and the effect of amyloid β-deposition on cognition are also highly dependent on the mouse genetic background, with some strains that are highly affected while others are resilient.^6,18^ Here, we report a pilot study showing that a similar effect may occur for a tau-related phenotype: propagation. Of interest, one of the strains used in the amyloid β study, the commonly used C57BL/6 strain (which is a frequent background strain in transgenic lines) was fairly resilient to amyloid β.^6^ In our current data, this strain is clearly resilient to tau propagation as well. These data reinforce the idea that building genetic risk factors into different strains may prove to be very fruitful.^19^

Since propagation rates may be related to the rate of clinical progression in humans with Alzheimer’s disease,^20,21^ these results may provide insight into further exploration of genetic underpinnings of Alzheimer’s and tauopathy symptoms. Some data already suggest specific genetic factors in this regard. The most common genetic risk factor for non-autosomal dominant Alzheimer’s disease, the *APOE e4* gene, has been associated with more severe tau-related neurodegeneration^22^ and clinical rates of progression.^23^*BIN1*, which is one of the most significant genetic risk factors for Alzheimer’s disease, has also been clearly linked as a modulator of tau pathology^24^ and an intermediate of tau pathology propagation.^25^ It seems likely that other genes, or combinations of genes, may impact various aspects of tau pathobiology as well.

In conclusion, these results suggest that the rate of tau propagation may depend, to some extent, on heritable factors. Understanding the genetic loci that impact the kinetics of tau aggregation, propagation and neurotoxicity may open new avenues towards therapeutics for tau-related neurodegenerative diseases. Moreover, a similar approach might detect strain-dependent differences in other misfolded protein disorders in which propagation has been implicated.^26^

## Supplementary Material

fcac048_Supplementary_Data

## Abbreviations

AAV =: adeno-associated virus
APOE =: apolipoprotein E
A/P =: anteroposterior
BIN1 =: bridging integrator 1
DAPI =: 4′,6-diamidino-2-phenylindole
D/V =: dorsoventral
eGFP =: enhanced green fluorescent protein
M/L =: mediolateral
