# Supplementary material for: Tau propagation is dependent on the genetic background of mouse strains
Source: Brain Commun. 2022 Feb 23;4(2):fcac048. doi: 10.1093/braincomms/fcac048 (PMC8952249; doi:10.1093/braincomms/fcac048)
Supplement: fcac048_Supplementary_Data [file fcac048_supplementary_data.pdf]

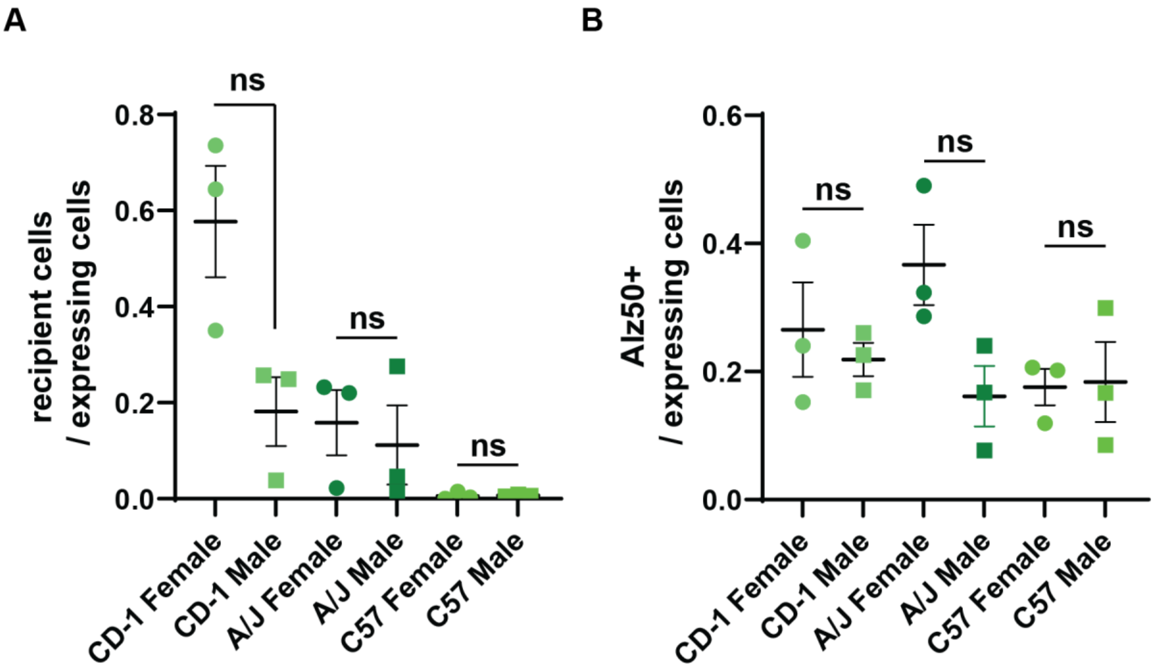

Supplementary Figure 1. A-Ratio of recipient cells (hTau+, eGFP-) to AAVs expressing cells (hTau+, eGFP+) in male versus female animals in the CD-1, A/J and C57 mouse strains showing a non-significant difference between males and females. **B-** Ratio of misfolded tau positive cells (Alz50+) to expressing cells (eGFP+) in male and female animals in the CD-1, A/J and C57 mouse strains showing a non-significant difference between males and females. N=3 per sex and strains, Mann-Whitney non parametric test was used to compare groups.
